# Corneal Innervation Research at a Crossroads: A Tool-Driven Roadmap for the Future

**DOI:** 10.1167/iovs.67.6.25

**Published:** 2026-06-15

**Authors:** Anna Matynia, Ian D. Meng, Brian M. Davis, Stephen C. Pflugfelder, Anat Galor, Victor L. Perez, Elizabeth R. Felix, Pedram Hamrah, Pantelimon Rompolas, Raul E. Ruiz-Lozano, Shamsuddin A. Bhuiyan, Kaveh Moghbeli, Evangelia Semizoglou, William Renthal, Harinder Singh, Jishnu Das, Marcin Golczak, Mary Ann Stepp, Catherine W. Morgans, Cintia S. de Paiva, Carl Y. Saab, Igor Spigelman, Rony R. Sayegh, Tally M. Largent-Milnes, Hao Wu, Wenqin Luo, Sreya Mitra, Heesuk Yoo, Sina Farsiu, Sejiro Littleton, Eden Jacob, Matthew T. McPheeters, Maryse Lapierre-Landry, Jordan J. Smith, Xi Wang, Roxana Florea, Made Airanthi K. Widjaja-Adhi, Scott Holmes, Zane Zemborain, Kate E. T. Laise, Nicholas J. Pondelis, Ki-Soo Jeong, Rui Chen, Michael W. Jenkins, Vivian Lee, Eric A. Moulton, Deborah S. Jacobs, Sue A. Aicher, Anthony J. St. Leger, Daniel R. Saban

**Affiliations:** 1 University of Houston, Houston, Texas, United States; 2 University of New England, Biddeford, Maine, United States; 3 University of Pittsburgh, Pittsburgh, Pennsylvania, United States; 4 Baylor College of Medicine, Houston, Texas, United States; 5 University of Miami, Coral Gables, Florida, United States; 6 University of South Florida, Tampa, Florida, United States; 7 University of Pennsylvania, Philadelphia, Pennsylvania, United States; 8 University of Illinois Chicago, Chicago, Illinois, United States; 9 Harvard Medical School, Boston, Massachusetts, United States; 10 Case Western Reserve University, Cleveland, Ohio, United States; 11 George Washington University, Washington, D.C., United States; 12 Oregon Health & Science University, Portland, Oregon, United States; 13 Cleveland Clinic, Cleveland, Ohio, United States; 14 University of California, Los Angeles, Los Angeles, California, United States; 15 University of Arizona, Tucson, Arizona, United States; 16 Duke University, Durham, North Carolina, United States; 17 University of California, Irvine, Irvine, California, United States

**Keywords:** corneal trigeminal innervation, OMICS, IVCM, retrograde labeling, animal models

## Abstract

Corneal innervation research has faced long-standing clinical challenges that only recent technological breakthroughs now make tractable. Advances in single-cell analysis, viral vectors, clinical imaging, and artificial intelligence provide integrated approaches for uncovering molecular mechanisms underlying functional outcomes and facilitating clinical applications. The National Eye Institute's U01-funded consortium on ocular surface innervation addresses major knowledge gaps in characterizing corneal-projecting neurons, understanding neuroimmune and epithelial interactions in the cornea, and translating animal model findings to human pathology. The unique properties of the cornea (transparent, avascular, and densely innervated) make it ideal for neurobiology research on peripheral and central sensory processing. Through a multi-institutional collaboration, the consortium roadmap systematically integrates four complementary pillars: retrograde labeling, omics technologies, animal models, and research models and human clinical imaging. The roadmap progresses through three phases: standardizing methodological foundations, achieving technological convergence through multimodal synthesis, and advancing clinical translation via cross-species harmonization, therapeutic target identification, and patient characterization strategies. This coordinated approach transforms isolated findings into mechanistic frameworks, demonstrating how technological convergence combined with collaborative science accelerates discovery and establishes foundations for understanding ocular surface circuitry in physiological and pathophysiological contexts, including pain.

In 2010, the National Eye Institute (NEI) Office of Program Planning and Analysis convened a multidisciplinary workshop that identified critical research gaps and scientific opportunities in ocular pain and sensitivity. The workshop highlighted that debilitating ocular sensations substantially decrease patients’ quality of life by interfering with activities of daily living, yet options for effective treatment and long-term relief remained severely limited. Workshop participants emphasized the need for “a full understanding of the primary afferents innervating ocular structures, including characterization of the heterogeneity of fibers and function,” called for new imaging systems and software to delineate corneal innervation patterns, and recognized that the roles neurons play in corneal health and disease—particularly the molecular and cellular mechanisms responsible—have yet to be determined.

Only recently has the technological landscape transformed dramatically enough to address these gaps. Advances in single-cell analysis, viral vectors, artificial intelligence (AI), and clinical imaging now make possible the integrated approaches necessary for bridging molecular mechanisms with functional outcomes and clinical applications. Recognizing these technological advances, the NEI launched the Anterior Segment Initiative (ASI) in 2021, culminating in the U01 funding opportunity: “Ocular Surface Innervation from Cell Types to Circuit Function.” This initiative specifically targeted the research gaps identified in 2010, establishing a consortium approach focused on corneal and ocular surface innervation as the most tractable entry point for understanding anterior segment neurobiology.

This white paper presents a consortium roadmap structured around four technological pillars: (1) retrograde labeling for target-specific neuronal identification; (2) omics technologies for molecular characterization; (3) animal models for mechanistic studies; and (4) clinical imaging for translational applications. This roadmap projects future directions based on the practical experience of consortium members actively working across these pillars; it is not a report of original findings or a catalog of planned experiments. Most importantly, it is the systematic integration of these pillars—not the individual technologies alone—that creates capabilities impossible through isolated approaches. The roadmap progresses through three phases: establishing reproducible methodological foundations, achieving technological convergence and multimodal synthesis, and advancing toward precision medicine through cross-species harmonization, therapeutic target identification, and patient characterization strategies.

This systematic integration represents a shift from descriptive studies toward mechanistic understanding of corneal innervation function and dysfunction. The consortium's coordinated application of convergent technologies directly targets research gaps that have persisted for decades, positioning the field to transform fragmented knowledge into mechanistic frameworks guiding therapeutic development. This consortium model demonstrates how coordinated multi-institutional integration can accelerate discovery beyond what individual technologies achieve separately, providing a template for addressing complex biological systems that require coordination across molecular, cellular, circuit, and imaging at the basic and clinical levels. Ultimately, these efforts will establish new opportunities for treating corneal disease, including intractable neuropathies that remain poorly understood and difficult to manage.

## Corneal Circuitry and Neuroimmune Networks

The cornea represents a remarkable evolutionary solution to the challenge of maintaining transparency while providing robust environmental protection. The complexity of the cornea results from adaptations that produced a densely innervated, stratified non-keratinized epithelium, stroma, and endothelium lacking lymphatic and blood vessels. These unique properties allow the cornea to be transparent while being continuously exposed to the environment with only a tear film separating it from a broad range of potentially damaging mechanical, thermal, and chemical irritants and infectious agents. Evolution has solved the problem of homeostatic regulation of this vulnerable structure by combining protective reflexes (blinking, tearing) with cornea-specific immune components including immune privilege,^1^ neuroimmune interactions, keratocyte–nerve interactions involved in both homeostasis and injury repair,^2^^–^^4^ and epithelial–nerve interactions^5^ that regulate epithelial cell division and injury responses.^6^^,^^7^ This consortium leverages recent technological advances to address longstanding gaps in understanding how corneal innervation maintains tissue homeostasis, processes sensory information, and responds to disease through coordinated neuroimmune and neuroepithelial interactions.

**Figure 1.**
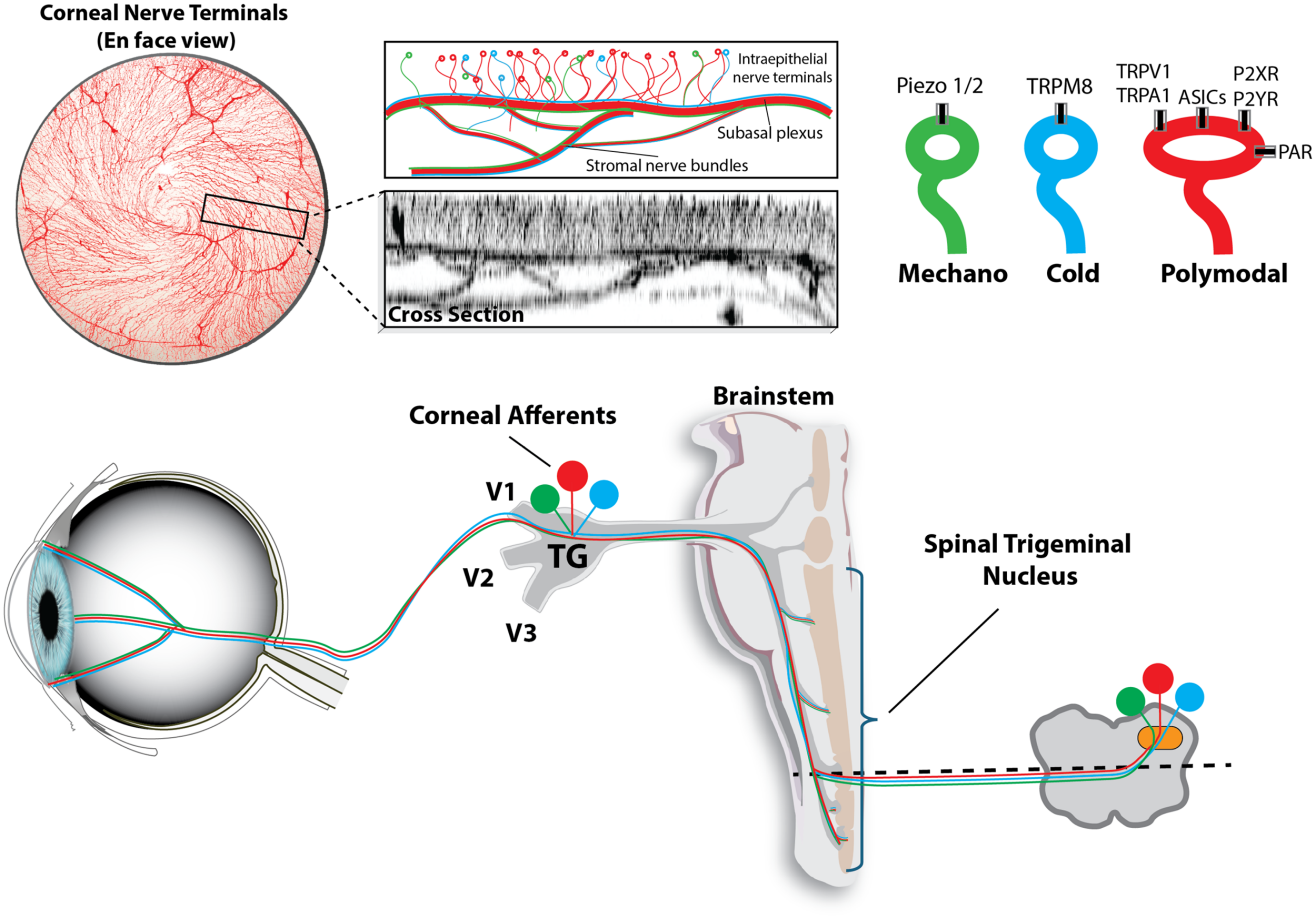
**The ocular surface is densely innervated by sensory corneal afferents.** These axons enter the corneal stroma as thick nerve bundles that branch into fine radial processes forming the subbasal nerve plexus. Individual nerve terminals traverse Bowman's layer and extend into the intraepithelial space, ultimately reaching the superficial layers of the corneal epithelium. The cell bodies of these corneal afferents reside within the TG, specifically in the ophthalmic branch, which together with the maxillary and mandibular branches constitute the three major divisions of the trigeminal system. The spinal trigeminal nucleus represents the next stage of the sensory pathway, where central processing of corneal afferent input occurs.

All corneal sensory innervation originates from pseudounipolar neurons within the trigeminal ganglion (TG), hereafter referred to as “corneal afferents” (Fig. 1), that developmentally derive from the neural crest and trigeminal placode.^8^^,^^9^ The TG contains three distinct clusters forming the major branches: V1 (ophthalmic nerve innervating the eye and upper face including corneal nasociliary fibers), V2 (maxillary nerve), and V3 (mandibular nerve). Although the cornea is considered to be innervated by the V1 branch, a case report in a human and studies in non-human primates indicate that corneal afferents are also located in the V2, although the extent and consistency are not fully known, and this innervation has not been identified in rodents.^10^^–^^14^ The cornea is considered the most densely innervated tissue in the body:^15^ The central cornea contains over 5000 nerve terminals per square millimeter in rats,^16^ 3000 in mice,^17^ and over 7000 in humans.^18^ Despite this high innervation density, retrograde labeling has revealed that these extensive terminal fields correspond to only 200 to 260 neuronal cell bodies within the TG.^18^ This remarkable convergence highlights the extensive branching patterns of individual corneal projecting nerves and underscores the precision required for their identification and functional characterization. Corneal innervation is comprised primarily of sensory nerves, but variable contributions from sympathetic and parasympathetic nerves are species dependent.^13^^,^^14^^,^^19^

Corneal afferents comprise three functionally distinct populations based on electrophysiological properties and stimulus responsiveness (Fig. 1).^20^^–^^24^ Mechanoreceptors respond primarily to mechanical stimulation through myelinated Aδ fibers, expressing mechanosensitive PIEZO1/2 channels that detect tissue deformation and contribute to protective blink reflexes. Polymodal nociceptors respond to mechanical, thermal, and chemical stimuli via unmyelinated C-fibers, and these neurons express diverse channel repertoires including temperature-sensitive transient receptor potential (TRP) channels (TRPA1, TRPV1, TRPM8), acid-sensing ion channels (ASICs), mechanosensitive PIEZO1/2 channels, purinergic receptors (P2XR, P2YR),^25^^,^^26^ and protease-activated receptors (PARs)^27^ that collectively shape nociceptor activation and sensitization. Cold receptors activate in response to cooling, with 20% to 50% exhibiting paradoxical heat responses. These cold-responsive corneal neurons subdivide into low-threshold populations that drive basal tearing in response to mild evaporative cooling^24^^,^^28^^,^^29^ and high-threshold populations that contribute to noxious reflex tearing and irritation triggered by severe corneal dryness.^21^^,^^28^^–^^33^ These differential functions are maintained at the circuit level through distinct projection patterns within the spinal trigeminal nucleus.

Central processing of corneal afferent inputs occurs within distinct rostral (anterior) and caudal (posterior) regions of the spinal trigeminal nucleus (Vc). Both regions respond to mechanical and TRP channel stimulation of the ocular surface, with different proportionality.^34^^–^^37^ Studies using stimulating electrodes or immediate early gene activity analyses identified connectivity between the Vc and the parabrachial nucleus, with the potential for differential support of reflex tearing and blinking versus sensory-discriminative and affective-motivational aspects of pain processing.^35^^,^^36^ This anatomical organization between the anterior and/or posterior Vc regions to the brainstem, thalamic, and/or parabrachial recipient areas including ipsilateral and contralateral connectivity bears further exploration; by doing so, we will gain better understanding of the foundation for both protective reflexes and conscious pain perception. Further investigation of these circuits will help explain how corneal pathology can simultaneously disrupt homeostatic functions and generate complex symptom profiles, including persistent pain.^34^^–^^36^

**Figure 2.**
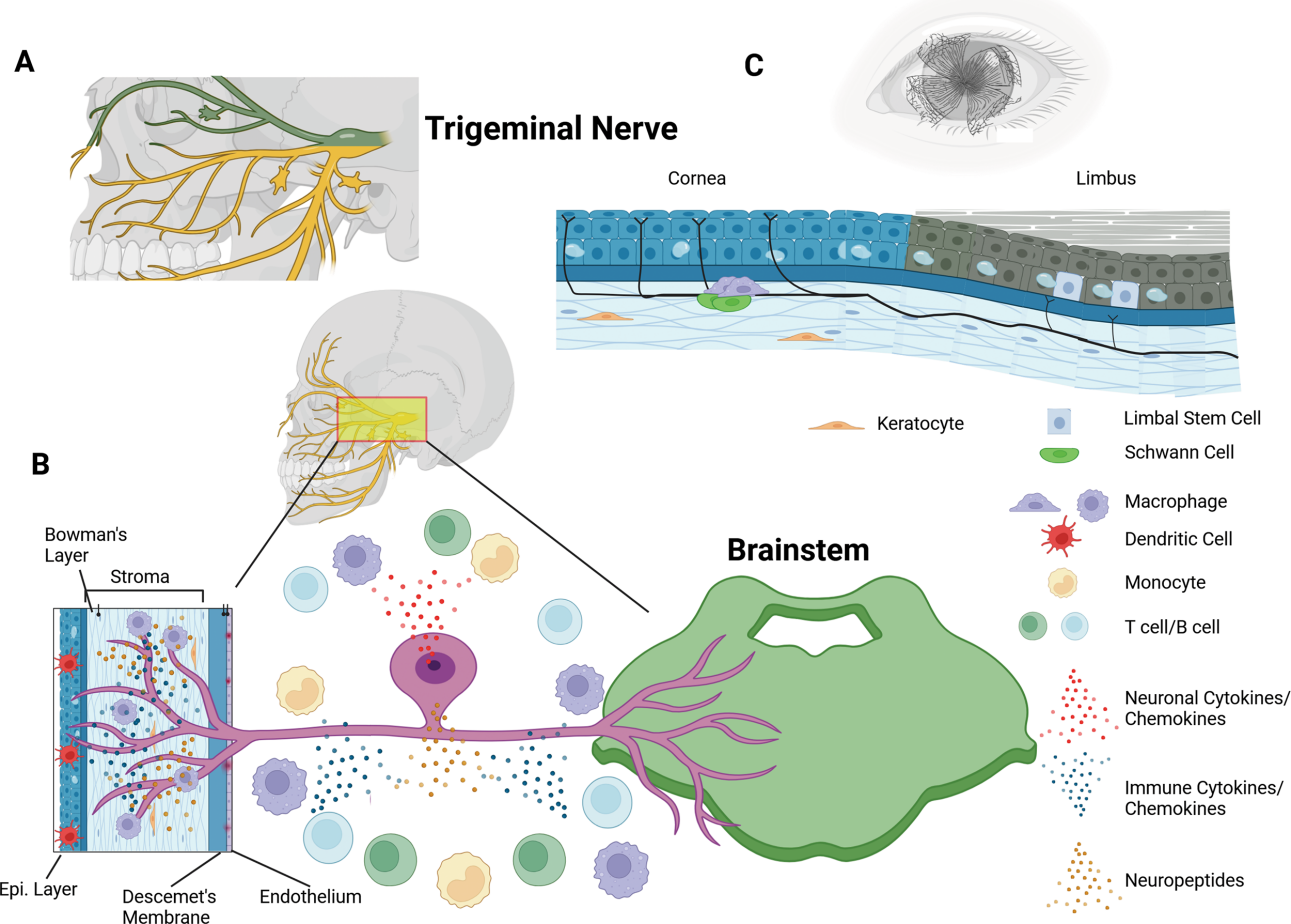
**Neuroimmune interactions influence corneal homeostasis.** (**A**) Cell bodies for corneal projecting afferents (*green*) reside in the TG, where they make up less than 1% of all neurons in the TG. Other neurons (*yellow*) project to other areas of the face. (**B**) Conceptualization of communication between the cornea and trigeminal brainstem as peripheral and central targets, respectively, without synaptic-specific localization. Within the TG and the cornea, immune cells, keratocytes, and nerves are in constant communication, each with the potential to produce cytokines, chemokines, and neuropeptides. Disruption of these communication networks through trauma, DED, and/or infection can result in alterations in sensation at the surface and in immune responses at the cornea, TG, and trigeminal brainstem. (**C**) Neuroimmune interactions also contribute to the unique innervation patterns in the cornea. As corneal nerves enter the corneal stroma from the limbal region, the nerve plexus forms the conventional “whorl” pattern (*top*). The unique “whorl” pattern is maintained by corneal immune and support cells (*bottom*) because depletion of these cells results in altered sensation and a loss of the “whorl” pattern. Depiction is generalized from primates.

Beyond supporting sensory and reflex functions, corneal innervation plays critical roles in tissue homeostasis through neuroimmune interactions (Fig. 2). Mounting evidence demonstrates bidirectional communication between sensory neurons and immune cell populations at both corneal and TG levels.^38^^–^^41^ The cornea harbors unique populations of innate immune cells that respond to infection and environmental insults, and the TG contains its own complement of immune cells that respond to these corneal events. Corneal afferents express cytokines and cytokine receptors that choreograph these immune responses,^42^^,^^43^ suggesting sophisticated coordination between proximal and distal neural and immune systems in maintaining corneal homeostasis.

The importance of these interactions is illustrated by recent preliminary studies identifying a tripartite cellular niche comprised of macrophages, nerves, and Schwann cells required for maintaining PIEZO2-mediated mechanosensation in corneal tissues.^44^ Another example occurs following injury or disease, when sensory fiber retraction is often accompanied by sympathetic postganglionic fiber invasion alongside blood vessels and lymphatics, with complete healing requiring sympathetic fiber withdrawal and sensory reinnervation.^45^^–^^50^ These dynamic interactions, combined with the accessibility and ease of evaluation of the cornea, position it as an exceptional model for understanding peripheral nerve regeneration, epithelial remodeling, and neuroimmune–glial–epithelial coordination.

To address these complex biological questions, this consortium integrated complementary approaches encompassing four technological pillars: retrograde labeling for target-specific neuronal identification, omics technologies for molecular characterization, animal models for mechanistic studies, and clinical imaging for translational applications. The core components that are highlighted in this paper emerge from multi-institutional partnerships and core principles of integration across laboratories, species, and methodologies.

## Retrograde Labeling and Other Neuronal Tracing and Targeting Methods

The ability to identify corneal-projecting neurons relies on retrograde labeling, where a tracer is introduced to the corneal afferents and transported back to the cell bodies of innervating neurons in the TG. Identifying the small portion of corneal projecting neurons (∼1%–2%)^13^^,^^14^^,^^19^^,^^51^ among the backdrop of those projecting to all other trigeminal structures requires clear and precise retrograde labeling. The value of retrograde labeling undoubtedly lies in its ability to provide spatial, temporal, and/or cell type specificity, with its greatest contribution arising through partnership with other technologies such as immunohistochemistry, electrophysiology, and sequencing to facilitate in defining neuronal function.

Traditional retrograde labels include wheat germ agglutinin and cholera toxin B (CTB) with histological detection methods that evolved from colorimetric tracers to those with intrinsic fluorescence (Fluorogold, Fast Blue) to fluorophore-conjugated compounds (Alexa, FITC, etc.) enabling multicolor labeling and enhanced co-labeling capabilities. These approaches have helped show that corneal innervation is comprised of primarily sensory nerves with variable contributions from sympathetic and parasympathetic nerves depending on species.^18^ Identified afferent types include polymodal nociceptors and cold-sensing transient receptor potential melastatin 8 (TRPM8)-expressing C-fibers, and mechanosensing PIEZO2-expressing Aδ neurons.^52^ In disease models, retrograde labeling has been essential for investigating changes in corneal afferents.^53^^–^^57^

Genetically encoded cell markers can also be used to label corneal-projecting TG neurons. Advanced approaches include conditional and modifiable transgenic mouse lines and viral gene delivery systems that provide enhanced capabilities for altering and/or recording gene and cell functions. Specific corneal projecting neuronal populations can be identified using Cre recombinase driven by cell-type-specific promoters in floxed reporter mice.^58^ Activity can be observed using cell-type-restricted genetically encoded calcium indicators (GCaMPs) or modified using some classical tracers such as FM1-43 which preferentially targets and blocks mechanosensitive PIEZO2 receptors via permeation, as shown using exogenous TRP or P2X_2_ channel recombinant expression systems.^59^^–^^64^ Viral gene delivery represents a powerful class of retrograde tracers, although best practices for corneal innervation remain limited.^65^ Improvements in spatial and cell-specific labeling and detection methods will facilitate discovery of differences in homeostasis and disease in cornea-innervating neurons. Disease-specific applications include simultaneous dual tracing from cornea and meninges to identify overlapping TG populations in migraine models,^66^ tracking corneal afferent changes in dry eye disease progression, and characterizing reinnervation patterns following infection.

### Knowledge and Technological Gaps

Fundamental limitations in current retrograde labeling approaches restrict their application to corneal innervation research. Most traditional small molecule tracers and dyes lack cell-type specificity, creating challenges for targeted experimental designs studying distinct corneal afferent populations. Conditional knockout mice, small molecule labels, reporters, or GCaMPs are often limited by a lack of spatial precision if expressed throughout the animal, and each delivery method carries inherent trade-offs. For example, topical tracer applications often require corneal epithelial disruption, intrastromal injections create limited stromal scarring, and iontophoretic delivery causes temporary epithelial damage.^67^ Viral approaches present the opportunity to combine cell-type specificity with spatial restriction through a combination of viral tropism and strategically selected mouse lines, after serotype specificity, promoters, and cargo are better characterized.

A critical gap exists in standardized criteria for achieving successful retrograde labeling across studies. For example, the number of afferents that can be identified varies depending on the tissue preparation.^51^ Evidence-based guidelines for the selection of retrograde labels, delivery methods, tissue preparations, and image acquisition and analysis will allow selection of the best methods for the questions at hand, avoiding inconsistency across labs, methods, and species. These standardization challenges are particularly relevant in disease models where damaged tissue may take up labels differently from healthy tissue.^68^ Standardization of methods and analyses across research groups and disease models (migraine, dry eye, and viral keratitis) will facilitate cross-study comparisons of difficult-to-interpret pathological changes and therapeutic interventions.

The invasive nature of retrograde labeling creates a significant translational gap, as its use is restricted to animal models and requires inference for human applications. Most studies use rodent models, and validated approaches for larger species, such as non-human primates, are notably absent despite their potential to provide valuable translational insights. Closing this gap by linking precise retrograde labeling in animal studies to human biology may be possible by identifying shared transcriptomic signatures, although direct human validation remains challenging.

Last, most current retrograde labeling techniques primarily provide endpoint evaluations, creating a critical gap in understanding the temporal dynamics of corneal innervation. This limitation hampers studies of degenerative and regenerative processes that require longitudinal tracking of corneal afferent populations during disease progression, injury response, and recovery phases.

### Future Outlook

Systematic optimization and standardization represent immediate priorities for advancing retrograde labeling applications. Clear metrics are needed that will inform future study design on best practices for each molecular retrograde label (Fast Blue, CTB) and labels with modifying capabilities (adeno-associated virus [AAV], including retrograde vectors) through systematic comparison of titers and tropism, delivery methods (topical, intrastromal, iontophoretic), and tissue preparation approaches (in vivo, fixed tissue sections, cleared wholemount tissue). The goal of this endeavor is to guide informed choices to increase rigor and reproducibility and to allow cross-study comparisons whether the scientific questions require reliable yet sparse labeling for circuit tracing or dense labeling that is suitable for global analyses.

Activity-dependent neuronal labeling represents an emerging alternative to traditional retrograde approaches for identifying functionally relevant corneal afferents and central pathways. Live imaging of *corneal afferents* with a genetically encoded calcium sensor (GCaMPs) combined with receptor-specific ligands (e.g., menthol for TRPM8 cold sensory neurons) provides the precision to query specific sensory pathways in disease models of sensitization and degeneration/regeneration, in real time. Stimulus-activated neuronal capture through inducible Fos–targeted recombination in active populations 2 (TRAP2) systems would enable functional mapping of *corneal-responsive circuits*, identifying which neurons are active during specific stimuli or disease states.^69^ Although these approaches provide endpoint evaluations, they can be combined with longitudinal imaging approaches to link neuronal identity with temporal dynamics. Additionally, unexpected discoveries from transgenic approaches are revealing novel labeling patterns that challenge traditional assumptions. For example, preliminary studies show that Cx3cr1-Cre lines, originally designed to label macrophages, specifically target a subset of PIEZO2-expressing mechanosensitive neurons, suggesting developmental connections between neuroimmune populations that could inform both neuronal identification strategies and our understanding of neuroimmune interactions in corneal homeostasis and disease.^44^

Advanced viral delivery systems offer expanding capabilities for manipulating and monitoring corneal nerve function. AAV-delivered optogenetic and chemogenetic tools will enable precise temporal control of corneal afferent activity, and GCaMP will provide real-time activity monitoring in longitudinal studies. Together, these tools can overcome significant spatiotemporal and cell-type limitations. These approaches facilitate coordinated structure–function relationship studies informing disease onset and therapeutic target identification, in addition to providing some of the first anatomical cellular maps of sensory and cognitive aspects of pain.

The integration of optimized retrograde labeling with spatial omics, advanced imaging, and computational approaches will generate comprehensive maps of corneal innervation from molecular to circuit levels. Through systematic standardization and integration with emerging technologies, these approaches will provide the precision necessary for understanding corneal nerve function in health and disease, ultimately informing therapeutic strategies for corneal neuropathies and ocular surface disorders with the potential for viral delivery of therapeutics.

## Omics Approaches

Early anatomical studies across species categorized TG neurons based on soma size, myelination, and innervation targets, and electrophysiological studies differentiated neuronal subtypes by axonal conduction velocity and receptive field properties. Molecular markers, when used, were largely constrained to immunohistochemical assays for canonical markers such as neurofilament or calcitonin gene-related peptide (CGRP). With the advent of transcriptome-wide profiling technologies, particularly at the single-cell level, a new approach for categorizing cell types has emerged. Transcriptomically identified cell types are operationally defined as a group of cells that exhibit greater intragroup similarity in gene expression patterns than to other groups of cells.^70^^,^^71^ This definition of “cell type” is sensitive to technical and analytical choices, such as the gene expression patterns selected for defining similar groups of cells and clustering parameters. For these reasons, transcriptomically defined cell types are best supported when there is clear in vivo evidence that ascribes to them a distinct structure or function.

In the TG, efforts to profile neuronal and non-neuronal cell types have largely been performed in mouse, rat, and human using bulk and single-cell RNA profiling methods.^72^^–^^76^ As ganglia are predominantly composed of non-neuronal cells, methods to improve the representation of neuronal cells have focused on optimized nuclear isolation gradient protocols and/or fluorescence-activated nucleus sorting, followed by downstream single-cell sequencing protocols, or enzymatic whole cell tissue digestion.^72^^,^^73^^,^^75^^,^^77^^–^^85^ Recently, laser capture microdissection (LCM) was implemented to collect human dorsal root ganglion (DRG) neurons from sections for RNA sequencing,^86^ a method equally amenable for human TGs to gain near whole-cell transcriptomic insight from frozen tissues with spatial context.

The rapid pace of development of single-cell and spatial transcriptomic technologies has been coupled with advances in computational tools for interpreting the resulting high-dimensional data. Benchmarking studies have compared multiple available tools and found that optimal performance requires tailoring the tool to the properties of the data and the specific biological question. For example, one study systematically evaluated methods for mitigating batch effects across libraries and concluded that Harmony, LIGER, and Seurat 3 performed best across most metrics of batch-effect removal.^87^ In contrast, other studies focused on the integration of single-cell RNA sequencing (scRNA-seq)/single-nucleus RNA sequencing (snRNA-seq) data across species^88^ and found that scVI, scANVI, and Seurat 4 most effectively clustered transcriptomically similar cells within the mammalian phylogeny. Similarly, in cross-species TG work, authors observed that Seurat 4 and scVI consistently aligned homologous TG neuronal cell types between human and mouse datasets.^89^

In addition to the single-cell technology, sequencing depth, and computational tools, there are numerous downstream analytical decisions that often contribute to variability in cell type annotation across studies. Together, these challenges have led to growing interest in reference atlas construction to allow for more consistent and scalable annotation strategies. Toward this goal, recent cross-species work has integrated data from eight studies across mouse and human trigeminal and dorsal root ganglia^89^ (harmonized.painseq.com), identifying 15 neuronal and 11 non-neuronal populations that are conserved between mouse and human. In parallel, the authors constructed a DRG atlas and identified three additional neuronal subtypes absent from the TG: *Pvalb*+ A-fiber proprioceptors and two uncharacterized C-fiber populations marked by *Calca+Dcn* and *Rxfp1*. The non-neuronal subtypes were largely consistent between the DRG and TG; however, oligodendrocytes and astrocytes appeared uniquely in the TG, likely due to sequencing portions of the root entry zone where myelination transitions from Schwann cells to oligodendrocytes. Meningeal fibroblasts were also observed in the TG but not in the DRG.

### Knowledge Gap

Previous studies have transcriptomically phenotyped sensory neurons globally in the mouse TG without determining gene expression profiles for corneal-projecting afferents specifically.^72^^,^^81^^,^^85^ The TG houses a myeloid-enriched immune cell population^84^ that responds to corneal pathology,^90^ but the relationship between tissue-specific neuronal identity and local immune environments remains poorly understood. Advances in high-throughput multiomic profiling, cellular labeling, and spatial technologies now enable deep characterization of neurons within target organs while simultaneously profiling their associated immune microenvironments. Multiomic, tissue-specific datasets linking neuronal gene expression to anatomical locations and immune contexts will provide fundamental building blocks for understanding how neurons regulate corneal homeostasis and disease.

A critical gap that omics approaches can address involves understanding how cellular niches maintain specialized sensory functions in corneal tissues. Computational tools including receptor–ligand analysis (CellChat,^78^^,^^91^ CellPhoneDB,^92^ NicheNet^93^) and gene pathway enrichment analysis (Gene Ontology,^94^ Kyoto Encyclopedia of Genes and Genomes,^95^ Reactome^96^) provide frameworks for unraveling heterotypic cellular interactions and identifying key signaling pathways. There is a growing appreciation for the role of macrophage and dendritic cell–nerve interactions in maintaining somatosensory populations in normal corneal innervation and diseased states including diabetic neuropathy and wound healing.^40^^,^^44^^,^^97^^–^^99^ However, the underlying mechanisms in these respective states remain poorly understood, particularly with regard to how myeloid cells transition from homeostatic to pathological functions. The dense innervation and unique tissue architecture of the cornea position it as an accessible model for applying omics approaches to investigate these neuroimmune coordination mechanisms during corneal disease and regeneration.

The abundance, complexity, and diversity of neuroimmune–epithelial interactions in corneal homeostasis and disease are increasingly recognized,^5^^,^^100^^–^^105^ yet omics characterization of these three-way interactions remains incomplete. The cornea represents a unique epithelial tissue densely innervated by TG sensory neurons yet devoid of lymphatics and vasculature except in pathological responses, perhaps explaining why corneal afferents are crucial for tissue homeostasis, particularly epithelial maintenance.^106^ Spatial transcriptomics and multiomic profiling can reveal how gene expression patterns in sensory neurons, immune cells, keratocytes, and epithelial cells coordinate during health and disease. Recovery from corneal disease likely requires reprogramming intercellular communications among these four cell populations. The myeloid-predominant corneal immune milieu becomes perturbed during disease, with myeloid–nerve interactions implicated in pathological responses.^40^^,^^99^^,^^107^ We additionally note that, although the cornea is normally devoid of blood vessels, CD4^+^ T cells and macrophages infiltrating inflamed corneas produce vascular endothelial growth factor A (VEGF-A) and VEGF-B that can stimulate growth of both blood vessels and sympathetic nerves or alternatively lead to sensory nerve degeneration into the cornea^46^^,^^101^; however, there is no direct interaction between the new vessels and nerves. Omics approaches are needed to systematically characterize how corneal sensory afferents regulate immune cell recruitment and phenotype at the ocular surface and to define molecular mechanisms underlying neuroimmune–epithelial interactions in preserving homeostasis and responding to disease.

### Future Outlook

A critical future milestone is the development of harmonized and annotated atlases of corneal afferents. Current TG transcriptomic maps lack tissue-specific projection information, as they have not incorporated retrograde labeling to identify neurons based on their innervation targets. Combining retrograde tracing with single-cell transcriptomics will enable the creation of target-specific neuronal atlases that map gene expression patterns to anatomical projection sites. Multiomic approaches that simultaneously capture transcriptomic, epigenetic, and proteomic information from the same cells will provide even richer characterization of neuronal identity and functional states, revealing regulatory networks that control tissue-specific innervation patterns and responses to environmental perturbations. These atlases will clarify how corneal afferents differ transcriptomically from those innervating other trigeminal regions, providing insights into tissue-specific sensory and homeostatic functions. These integrated approaches will generate high-dimensional taxonomies of corneal afferent subtypes including PIEZO2^+^, TRPV1^+^, TRPM8^+^, neurofilament heavy chain (NFH)^+^, and somatostatin (SST)^+^ populations in health and disease states, with applications ranging from migraine-associated trigeminal sensitization to dry eye–induced corneal afferent remodeling and herpes simplex virus type 1 (HSV-1)-mediated denervation patterns.

Understanding the spatial organization of neuronal cell types within the TG and target tissues is essential for deciphering neural control mechanisms. The TG exhibits unique somatotopic organization, with neuronal cell bodies located in specific branches (V1, V2, or V3) based on their projection targets. Ongoing efforts correlating physiological responses of mouse TG neurons to their transcriptomic profiles through in vivo calcium imaging and retrograde labeling will continue, enhanced by evolving spatial transcriptomic technologies^108^^,^^109^ that enable high-resolution molecular profiling to map transcripts, proteins, and other biomolecules directly within intact tissues.^110^^–^^113^ A diverse array of platforms now enables molecular mapping across different scales and modalities (Table 1), although challenges remain (Table 2). These data from corneal tissue will transform our understanding by mapping the distribution of immune cells, epithelial cells, and stromal cells and revealing how these populations interact with nerve terminals under normal and inflammatory conditions.^114^^,^^115^ By integrating tissue maps with retrograde labeling studies that link corneal-projecting neurons to their TG transcriptomic profiles, we can infer which nociceptor subtypes innervate specific corneal epithelial and stromal layers. These approaches may identify new glial and immune cell subpopulations that modulate neuronal excitability and carry transcriptional signatures linked to neuroprotective or proinflammatory functions.^7^^,^^116^ Combined with perturbation studies, these methods will be essential for delineating complex bidirectional neuroimmune communications between the TG and target tissues and their roles in homeostasis and disease.

**Table 1.** Spatial Omics Technologies and Capabilities

Technology	Platforms	Capabilities	Resolution	Refs.
Spatial transcriptomics (targeted)	Xenium, CosMx, MERSCOPE	Hundreds to thousands of targeted transcripts	Subcellular	228–230
Spatial transcriptomics (whole)	Visium, Stereo-seq, GeoMx	Whole-transcriptome profiling	Tissue level	231–233
Spatial proteomics	PhenoCycler, GeoMx DSP	Multiplexed protein mapping	Variable	233, 234
Multiomics	GeoMx, CosMx	Simultaneous RNA and protein analysis	Tissue/subcellular	235
Open-source	IBEX community	Validated immunolabeling protocols	Variable	236
3D reconstruction	Volumetric imaging, serial reconstruction	Deep tissue architecture analysis	3D tissue level	237, 238

Technologies vary in resolution (subcellular to tissue level), throughput (targeted vs. whole transcriptome), and modality (RNA, protein, or multiomic). Platform selection depends on research questions, tissue characteristics, and analytical goals.

**Table 2.** Current Challenges in Spatial Omics and Computational Solutions

Challenge	Description	Solution	Refs.
Cell segmentation	Boundary detection in corneal/TG tissue architectures	Deep-learning segmentation algorithms	239
Multicellular deconvolution	Visium spots contain multiple cells	Single-cell reference-based deconvolution methods	240
Data integration	Lack of frameworks for cross-study comparisons	Harmonization tools, standardized atlases	241, 242
Spatial–molecular mapping	Distance between TG cell bodies and corneal terminals	Retrograde labeling combined with spatial profiling	147
User accessibility	Need for bioinformatics expertise	User-friendly interfaces, automated pipelines	—
Network analysis	Complex multiomic, multi-compartment data	Machine learning approaches (SLIDE, MIRA, CellOracle, CellPhoneDB, CellChat)	91, 92, 243–246

Challenges specific to corneal innervation research include cell segmentation in unique tissue architectures and spatial–molecular mapping across distant anatomical compartments (TG to cornea).

Retrograde labeling-defined corneal afferent populations in animal models will be systematically compared with human single-cell TG datasets to identify conserved “corneal molecular signatures” enabling inference of corneal afferent identity in human populations, bridging precise animal studies with clinical applications and therapeutic development. Tying molecular patterns to function through these comprehensive atlases will guide hypothesis-driven interventional studies, accelerate discovery of novel biomarkers and therapies, and ultimately revolutionize our understanding of neural circuits maintain ocular surface homeostasis, opening new avenues for precision ophthalmic medicine.

## Animal Models

Animal models of corneal, ocular surface disease, damage, or injury provide essential platforms for understanding mechanisms underlying corneal function including pain, epithelial barrier disruption, inflammation, nerve damage and regeneration, and nociceptor sensitization. Many of these can be evaluated at an anatomical level, in vivo with tear breakup times and corneal fluorescein staining, or ex vivo in corneal tissue with immunofluorescence or immunohistology. Evaluation at the functional level uses both electrophysiological and behavioral approaches, as pain cannot be directly queried in animals. Electrophysiological recordings of dissociated cells as well as in vivo recordings have been a mainstay of characterizing changes in both the corneal afferents and in the downstream circuits, including identifying changes in excitability in disease models^53^^,^^54^^,^^117^ with the holistic functional readout of pain behaviors. Both behavioral and neurophysiological readouts should scale with stimulus intensity or injury severity and reverse with established analgesics, with the therapeutic goal to alleviate corneal pain while preserving homeostatic function.

Quantifying spontaneous or ongoing pain is of particular interest due to its translational value in modeling clinical symptoms. Key behavioral measures of pain states in many different injury models include reduced locomotor activity assessed through open-field assays and wheel running,^117^^,^^118^ although decreased activity can also signify increased anxiety, requiring careful interpretation. Facial expression evaluated using rodent Grimace Scales converts facial action units into composite nociception scores,^119^^,^^120^ with orbital tightening as a robust indicator for eye pain.^121^^–^^123^ Injury-tending behaviors including escape responses and injury-site-specific attention provide additional pain measures. For example, in cornea and ocular injury, reflexive pain responses of spontaneous or evoked pain use measures of pain such as blink rate changes and eye rubbing, observed across species,^124^^,^^125^ that are unprovoked or elicited by mechanical stimulation, respectively. Cochet-Bonnet esthesiometry or von Frey filaments predominantly target myelinated Aδ fibers but also C-fibers,^124^^,^^126^^–^^130^ and chemical stimulation with hypertonic saline, capsaicin, or other TRP channel ligands target unmyelinated C-fibers^124^^,^^131^^–^^134^ for evoked assessments. In addition, precisely delivered selective activation of TRPV1-expressing nociceptors, primarily C- and Aδ-fiber afferents, can be alternatively achieved via optogenetic approaches with the temporal resolution of milliseconds.^135^ Motivational dimensions include conditioned place aversion and preference paradigms. In conditioned place preference, subjects seek environments paired with pain relief, as demonstrated in a lacrimal gland excision model of dry eye disease using optogenetic inhibition of primary afferents.^117^ Pain relief can also be an intrinsic characteristic of the environment, as is the case with photoallodynia, a frequent complaint of patients after corneal injury, chronic eye pain, and dry eye disease. In animal models, photoallodynia is assessed through light-avoidance behaviors.^66^^,^^136^^,^^137^

These nonverbal behavioral measures have been validated in animal studies using analgesics and in human patient studies, particularly for nonverbal populations including infants, enabling crucial connections between animal mechanistic studies and clinical pain assessment.^138^^–^^142^ These self-report paradigms that rely on operantly conditioned behaviors, such as licking to receive water rewards, can be considered ethologically consistent with patients self-reporting their pain.^135^ In general, there is a high concurrence of behavioral testing in animal models and human quantitative sensory testing approaches, in which patients respond with verbal reports or changes in facial expression and muscle tightening measured to varying types and intensities of stimuli.^141^^,^^142^ The ability to use similar techniques across animal models and human patient populations enables crucial connections to be made between animal-model-derived mechanistic studies and patient reports of clinical pain symptoms (Tables 3, 4). Still, comprehensively capturing the multidimensional nature of pain encompassing spontaneous pain, evoked responses, and motivational components remains challenging.

**Table 3.** Evoked Sensation Assessments for Corneal Pain: Cross-Species Methodology

Modality	Clinical Significance	Human Methods	Animal Methods	Alignment and Gaps	Refs.
Visual photosensitivity	Photoallodynia suggests neural dysfunction; impacts quality of life	Focal graded light; pain/discomfort ratings; blink; facial expression	Ambient light box; time in chamber; number of crosses; blink; facial expression	Graded stimuli comparable. Gap: Need localized stimuli in animals	130, 247, 248
Mechanical sensitivity	Wind/pressure exacerbation common in clinical presentation	Graded air pulse (Belmonte) or filament (Cochet-Bonnet); pain ratings; blink	Graded filaments; blink; head withdrawal	Blink overlap. Gap: Self-report vs. reflex differences	249, 250
Warm/heat pain	Hyper- or hypo- sensitivity indicates neural dysfunction	Heated air or contact thermode; threshold, intensity ratings; blink	Capsaicin; eye wipes, blink rate, eye closure, grimace	Little overlap. Gap: Need graded heat in animals	251, 252
Cool/cold pain	Hyper- or hypo- sensitivity indicates neural dysfunction	Cooled air or contact thermode, menthol; threshold; ratings; blink	Cooled air, menthol; eye wipes; blink; palpebral fissure; eye closure	Temperature stimuli comparable. Gap: Validation of pupil/facial measures	54, 253, 254
Secondary hyperalgesia	Outside primary area indicates central nociceptive system dysfunction	Mechanical; cold, heat at periorbital and distant sites; pain ratings	Mechanical (von Frey); cold/heat at non-corneal sites; withdrawal; escape; grooming	Gap: Few ocular studies in animal models test distant sites for generalized hypersensitivity	255
Chemical sensitivity	Reflects altered nociceptor function	Graded irritants; pain threshold/severity	Capsaicin, acid; hypertonic saline; eye wipes; blink; grimace	Chemical overlap. Gap: standardize concentration–response	253, 256
					

Evoked assessments measure pain responses to controlled stimuli. Human methods rely on self-report; animal methods use reflexive/behavioral responses. Key gaps include cortical versus reflex differences and need for standardized protocols.

**Table 4.** Spontaneous Pain Assessment for Corneal Pain: Cross-Species Methodology

Pain Construct	Human Assessment	Animal Assessment	Alignment and Gaps	Refs.
Pain intensity/severity	Direct: Self-report (NRS, VAS) Behavioral: Facial expression, vocalization	Grimace scales; pain-tending/grooming (eye wiping)	Gap: No substitute for self-report; well-being more informative	257, 258
Well-being and affect	Questionnaires: SF-MPQ2, MPI, WHO-5, PHQ-9, GAD-7	Decreased reward (sucrose); decreased exploration (open field); conditioned place tests	Composite measures could proxy clinical assessments	259–261
Impact on visual activities	Self-report (OSDI) Behavioral: Not measured	Decreased light chamber time; increased eyelid closure	Gap: Track human time in light vs. dark, screen time, reading duration	262
Impact on physical activity	Self-report ratings Behavioral: Activity trackers, logs	Reduced activity (wheel running, open field)	May reflect affective aspects of pain more than direct pain interference on activity	155

Spontaneous assessments capture ongoing pain without external stimulation. A key gap is that there is no substitute for self-report in animals. Composite measures and well-being assessments may better capture chronic pain complexity. NRS, numerical rating scale; VAS, visual analog scale; SF-MPQ2, Short-Form McGill Pain Questionnaire 2; MPI, Multidimensional Pain Inventory; WHO-5, World Health Organization Well-Being Index; PHQ-9, Patient Health Questionnaire-9; GAD-7, Generalized Anxiety Disorder 7; OSDI, Ocular Surface Disease Index.

Many diverse animal disease models that were originally established to study corneal, ocular surface, and ocular adnexal disease, damage, or injury are now being utilized to understand the specific pathophysiological mechanisms underlying neuronal dysfunction and pain. Direct corneal injury models, including epithelial debridement with heptanol, alkali burns, or mechanical scraping that produce acute denervation and robust immune infiltration, have been used to examine corneal healing, reinnervation, and plasticity that may contribute to hyperalgesic priming or latent sensitization.^143^

A current area of focus is dry eye disease, encompassing both aqueous-deficient and evaporative settings. Examples of animal models include environmental desiccating stress combined with scopolamine to reduce lacrimal secretions,^144^^,^^145^ surgical lacrimal gland excision,^117^ naturally occurring dry eye in aged mice representing age-related changes, and genetically modified mice lacking metalloprotease-9 (MMP-9) a protein that is elevated in human dry eye tears. These models have been valuable for evaluating corneal barrier function, inflammation, nerve morphology, and disease-relevant transcriptional changes.^146^^–^^148^

Evaporative dry eye is modeled using meibomian gland dysfunction (MGD) settings. One of the most common forms of MGD is associated with chronic ocular surface inflammation and can be immune driven, and it is studied in the allergic eye disease model. Likewise, ocular graft-versus host disease in mice, also seen in humans, models immune-driven MGD.^149^ Genetic approaches to mimic aspects of MGD have also been established, notably with acyl-CoA wax alcohol acyltransferase 2 (AWAT2) knockout mice that lack the key enzyme producing wax esters comprising 40% to 50% of meibum.^150^^,^^151^

Beyond ocular surface diseases, additional models target specific pathophysiological mechanisms. Photoallodynia research employing stimuli that trigger the neurological phenomenon underlying migraine reveals light aversion and pain responses that mirror those seen in corneal injury patients and animal models.^66^^,^^137^ Neuropathic pain has been modeled using ciliary nerve ligation following lateral canthotomy, offering one of the first animal models to investigate this poorly understood and difficult to manage condition.^118^ Finally, pathogen-mediated models of corneal nerve pathology include HSV-1 keratitis, in which some viral strains produce mild keratitis while preserving sensory function, whereas others cause severe keratitis with sympathetic innervation loss. Interestingly, corneal sensory nerves retract following HSV-1 infection and are replaced with sympathetic nerves that cannot sense stimuli or control the blink reflex like the original sensory nerves.^152^

### Knowledge Gaps

Standardization and validation of pain-related behavioral endpoints remain inconsistent across disease models and research sites. Although some studies have employed comprehensive behavioral batteries, including ongoing pain measures (orbital tightening/palpebral aperture), evoked responses (mechanical and chemical), and motivational assays (light avoidance, conditioned place preference), many have relied primarily on acute nocifensive responses that inadequately capture the multidimensional sensory discriminative, affective–motivational, and cognitive domains of pain. For example, although many dry eye model studies have reported increased blinking, few models have examined broader behavioral characteristics such as spontaneous squinting, altered wheel running, and anxiety/depression-like behaviors.^117^^,^^153^^–^^155^ These assessments of pain behaviors commonly rely on subjective scoring and lengthy observations, which hinder reproducibility and limit analytical speed.^156^^,^^157^ Furthermore, pain should be recognized as a scale, and such stimulus–response functions should be reported where possible.^158^^–^^161^ To these ends, validation with benchmark analgesics and positive controls remains inconsistent, limiting translational value for drug screening applications.

Similarly, integration among behavioral, molecular, and neurophysiological approaches presents significant gaps, particularly in disease models. Although corneal afferents are classically defined by physiological response properties, their full molecular diversity and developmental origin as resolved by single-cell transcriptomics and alterations under pathophysiological conditions remain incomplete. These links among transcriptomic profiles, neuronal functional properties, and behavioral outputs have yet to be established and will be foundational for selective targeting of signaling pathways associated with distinct aspects of homeostatic regulation and corneal pain.

Major barriers to progress exist in translation between animal models and human pathophysiology. Not only have relatively few studies examined functional changes in corneal afferents across different disease models, but also fewer studies are able to relate animal findings to humans. Three clear examples are the lack of data correlating corneal nerve changes in animal models with human corneal nerve morphology observable through clinical in vivo confocal microscopy, cross-species harmonization of molecular and cellular data that currently lacks systematic approaches, and the limited alignment of central signaling pathways between species. Although alterations in corneal innervation have been demonstrated in various disease models,^140^ systematic investigations across different model types and correlations with specific functional alterations are limited by time-, subject-, and scientist-intensive requirements. Harmonized transcriptomics has yet to account for species differences in precise molecular mechanisms mediating corneal sensation and neuroimmune communication. Finally, animal studies of corneal sensory central processing are needed that will be mutually informative with human brain imaging to characterize distinct features of ocular pain, inflammation, and chronic dry eye across different diseases.^162^^–^^167^

### Future Outlook

Advanced behavioral quantification approaches will enhance the validation and drug screening capabilities of these models. Leveraging AI-assisted analysis, spontaneous and evoked corneal pain behaviors spanning sensory–discriminative, affective–motivational, and cognitive domains can be precisely measured in healthy and disease models. Such measurements trained with benchmark analgesics and validated against controls will add further rigor. For example, machine learning–driven pose estimation reduces observer bias and analytical time by providing rapid, automated labeling of behavioral blink reflex and identifying latent features within multimodal, high-dimensional datasets. This integration of comprehensive behavioral batteries with automated, reproducible analysis will reduce investigator bias and standardize assessments across research sites, facilitating comprehensive stimulus–response functional analyses and high-throughput screening of novel therapeutics.

To further refine advanced behavioral quantification, a molecular definition of the circuits generating ongoing and evoked corneal pain is essential. This definition can be achieved through opto- and chemogenetic manipulations of molecularly identified corneal afferents, paired with time-locked behavioral readouts and neural recordings. Together with the application of data-dense methods such as multi-electrode arrays, or recordings from dissociated corneal afferent and their recipient brainstem regions will help elucidate circuit plasticity in chronic pain states. Also, cross-validation with human functional imaging approaches will ensure translational relevance, allowing for selective interventions that enhance corneal healing and symptom resolution without disrupting ocular surface homeostasis.

A holistic approach incorporating animal models with well-characterized pain-related behavioral and electrophysiological endpoints, omics profiling to reveal mediators of neuro-surface crosstalk through candidate ligand–receptor pair identification, and integration of circuit-level data will provide researchers the ability to uncover conserved mechanisms and establish causal links between surface pathology and neuronal dysfunction. These integrated methodologies will close the gap between mechanistic insights from animal models and clinical applications, driving forward therapeutic innovations to improve analgesia, promote epithelial wound healing, and modify immune responses and healthy nerve regeneration for corneal pain and related ocular surface disorders.

## Clinical Intravital Imaging

In vivo confocal microscopy (IVCM) is widely used in clinical imaging studies of ocular innervation and disease, although no single imaging modality can fully capture corneal nociceptive biology and pathophysiology. IVCM is a real-time, non-invasive imaging technique based on conjugate alignment of light rays focused on and reflected from tissue, captured by an objective lens.^168^ The power of IVCM lies in its ability to image corneal structures at a near-histological level, with a lateral resolution of 1 to 2 µm and axial resolution of 4 µm.^169^ The high-resolution images produced by IVCM, with a typical field of view of 400 × 400 µm, enable visualization of corneal morphology from epithelium to endothelium, including innervation, immune cells, pathogens, and disease manifestations.^170^^–^^172^ This detailed anatomical window into corneal homeostasis and pathology has yielded substantial insights into the pathophysiology of multiple ocular surface disorders in both human and animal studies. Notably, IVCM imaging allows for clear quantification of corneal nerves, especially in the corneal subbasal nerve plexus.^172^

Clinically, IVCM is most frequently used to evaluate corneal nerve morphology in various ocular surface diseases, primarily to help differentiate between neuropathic corneal pain (NCP), a subtype of neuropathic pain localized to the cornea,^173^^,^^174^ and dry eye disease (DED),^175^^,^^176^ as multiple studies have suggested many patients treated for DED may instead have neuropathic pain.^168^ The differential diagnostics between neuropathic pain and DED are the clinical features including positive corneal fluorescein staining, decreased tear break-up time, and decreased tear volume that are generally present in DED but absent in neuropathic pain. However, when a patient presents with corneal pain and a lack of pathognomonic clinical findings, distinguishing between neuropathic pain and mild-to-moderate DED is challenging due to overlap in unpleasant or painful sensations.^169^^,^^176^^,^^177^ Even the use of advanced imaging techniques, such as IVCM, has not been able to fully resolve this overlap, as both have been associated with reduced subbasal nerve metrics, including corneal nerve fiber density, corneal nerve fiber length, and corneal nerve branch density, when compared to controls. However, several groups have recently suggested that microneuromas, detectable by IVCM, may serve as candidate biomarkers for NCP.^173^^,^^175^^,^^178^^,^^179^ Microneuromas are microscopic, abnormal formations of nerve endings at stromal or epithelial sites of damage where injured axons have failed to properly regenerate, leading to the formation of an irregularly shaped enlargement of the nerve fiber.^180^^,^^181^ The U.S. Food and Drug Administration recently approved microneuromas as a diagnostic criterion for the differentiation of NCP from DED in a clinical trial.^182^ This recent inclusion is clear, as a 2023 meta-analysis in eyes with DED compared with controls made no specific mention of microneuromas in DED, whereas decreased total corneal nerve fiber length, corneal nerve branch density, and corneal nerve trunk density were demonstrated.^182^^,^^183^ Beyond the work in clinical populations using IVCM, both DED and NCP are being modeled in animals using similar diagnostic criteria with the goal of improving understanding and classification schema for these disease entities (see Animal Models section).^184^^–^^189^ IVCM is also used clinically in the early detection of infectious keratitis, specifically fungal hyphae and *Acanthamoeba* cysts, where it achieves a faster time to diagnosis compared to ocular cultures.^190^ Further, IVCM has shown that corneal nerve loss may represent a surrogate biomarker for detecting diabetic peripheral and cardiac autonomic neuropathy,^191^^,^^192^ dementia,^193^ multiple sclerosis,^194^ and systemic autoimmune diseases.^195^

### Technical, Methodological, and Knowledge Gaps

Despite its high-resolution capabilities, IVCM in humans is constrained by several factors limiting clinical utility and research applications (Table 5). First, image acquisition protocols lack standardization. Studies have focused on different corneal regions (central, peripheral, or inferior whorl) with well-described morphological differences in innervation, making cross-study comparisons difficult.^196^ Terminology is also inconsistent: the complex spiraling pattern of the inferior whorl makes standard terms such as “trunk” and “branch” difficult to apply,^183^ and “nerve fiber density” variably describes either nerve count or total length per frame. Second, inter-individual variability is influenced by age, sex, diseases, medications, and environment, complicating interpretation due to lack of standardized normative data.^197^ Third, IVCM images only 0.16 mm^2^ at a time and cannot resolve suprabasal epithelial nerve terminals, limiting generalizability in heterogeneous diseases such as DED. Several groups have attempted to mitigate the limited IVCM field of view by combining morphological nerve metrics across multiple corneal regions.^198^^,^^199^ However, manual image selection may introduce sampling bias and consequently may not represent an appropriate estimate of global nerve parameters.^200^ Fourth, structural interpretation remains ambiguous without immunostaining. Microneuroma-like hyperreflective features are detectable in healthy corneas, but stromal nerves crossing Bowman's layer normally appear hyperreflective and dysmorphic.^201^^–^^207^ These normal features may be mistakenly attributed to pathology. Motility-based analysis also reveals that morphometric assessment alone has led to immune cell mischaracterization.^208^ Finally, manual analysis of large IVCM datasets is costly, time consuming, and prone to interobserver variability. These constraints have driven the adoption of technological solutions.

**Table 5.** IVCM Technical and Knowledge Gaps in Corneal Innervation Research.

Challenge Category	Key Issues	Solutions and Future Directions	Refs.
Standardization and normative data	• Inconsistent acquisition protocols and terminology across studies (central vs. peripheral vs. whorl) • Variable definitions (e.g., “nerve fiber density” = count vs. length) • Lack of normative reference data accounting for age, sex, disease, medications	• Protocol harmonization and consensus terminology • Standardized region-specific guidelines • Multisite normative databases with demographic stratification (phase 1)	183, 196, 197
Technical and resolution limitations	• Small field of view (0.16 mm^2^) with high local variability • Cannot yet image suprabasal nerve terminals, lack of depth imaging of stromal nerves • Structure ambiguity without immunostaining (microneuromas vs. normal features)	• Widefield imaging, automated mosaicking, and statistical sampling methods • Higher-resolution and depth-imaging technologies and multimodal integration (OCT, multiphoton) • Dynamic imaging for motility-based cell identification; AI classification	198–205, 209, 210
Analytical efficiency	• Manual analysis is time consuming, costly, and subject to interobserver variability • Difficult to achieve consistent cross-site comparisons	• AI/deep learning: CNNs for automated feature extraction and classification • Validated for diabetic neuropathy, infectious keratitis, DED, NCP	169, 171, 210–213
Neuroimmune interactions	• Cannot distinguish specific immune cell types by morphology alone • Unable to assess bidirectional TG-cornea neuroimmune networks	• Dynamic IVCM distinguishing mobile vs. stationary cells • Integration with spatial omics and retrograde labeling • Postmortem IVCM-immunostaining correlation	206
Peripheral–central integration	• IVCM limited to peripheral corneal nerves • Cannot assess central pain mechanisms, trigeminal pathway, or brain processing • Chronic pain involves both peripheral and central sensitization	• Integrate IVCM with MRI approaches: Diffusion MRI for trigeminal pathway microstructure fMRI of evoked pain-related brain activity Resting-state fMRI of spontaneous pain-related central mechanisms	163, 214–218, 263–265
Preclinical–clinical translation	• Gap between animal imaging (multiphoton, genetic tools) and clinical IVCM • No nerve fiber typing capability in clinical imaging • Limited longitudinal tracking	• Cross-species imaging harmonization • Optogenetics for function-correlated perturbation studies • Morphology-based fiber typing via antibody-validated approaches	219–225

IVCM faces challenges in standardization, technical capabilities, analysis efficiency, and functional/mechanistic assessment. Solutions leverage AI, multimodal imaging, spatial omics, and MRI integration.

Several technological and methodological advances promise to address current limitations and expand imaging capabilities for understanding corneal damage, degeneration, and pain.^171^^,^^173^^,^^190^^,^^209^^,^^210^ Advances in AI and deep learning offer solutions to these analytical limitations. Convolutional neural networks (CNNs) can effectively register as well as fuse IVCM images into larger field-of-view mosaics^211^ and extract features from IVCM images without human intervention,^212^ demonstrating evidence of potential for distinguishing diabetic peripheral neuropathy,^213^^,^^214^ infectious keratitis,^215^ and ocular surface diseases, including DED and NCP.^171^^,^^173^

Understanding neuroimmune interactions represents another critical frontier for IVCM. Although IVCM visualizes both immune cells and nerves, morphology-based identification has not yet been shown to distinguish specific immune cell types. Combining IVCM with postmortem immunostaining, as shown in paired optical coherence tomography (OCT)–histopathology retina studies, can address this limitation. Dynamic IVCM imaging also shows promise, distinguishing mobile stromal macrophages from stationary keratocytes based on movement patterns and enabling real-time functional assessment patterns.^208^ However, IVCM remains limited to corneal imaging and cannot assess broader bidirectional neuroimmune networks linking the TG to target tissues.

### Future Outlook

A significant gap exists in integrating peripheral nerve imaging with central nervous system assessment. Although peripheral innervation serves as the entry point for noxious stimuli transduction, central mechanisms that give rise to pain sensation represent the culmination of ascending and descending somatosensory processing. Moreover, by evaluating central mechanisms that give rise to pain perception, we can better understand the underlying pain modulatory processes and situations where pain is discordant with nociceptive stimulation. It will be imperative, as it relates to the intersection of pain and nociception, to develop methods using AI that integrate the magnitude of information available from clinical behavioral, and neuroimaging-based analyses. Chronic pain conditions involve both peripheral and central sensitization, yet the study of ocular pain in patients has been primarily focused on structural damage of peripheral nerves. By engaging modern neuroimaging techniques, practical approaches to directly study the structure and function of the peripheral and central nervous system in patients as it relates to pain are feasible, but integration of these approaches has thus far been limited.

Ultimately, ocular pain cannot be understood by examining only the ocular surface and peripheral nerves as the brain constructs the pain experience. Central nervous system imaging represents a critical frontier for understanding pain mechanisms beyond peripheral nerve assessment. In mice, circuit tracing and the Fos–TRAP system (see Animal Models section) is a singular endpoint method for anatomical and functional mapping. In addition, non-reflexive behavioral models allow mapping of sensory perception onto neural circuits in the brain using electrophysiology. In humans, magnetic resonance imaging (MRI) permits structural and functional assessment of the nervous system beyond corneal sensory afferents. High-resolution T1 MRI quantifies regional volumetric changes in brain structure in pathological conditions, and diffusion imaging allows tracking of the trigeminal cranial nerve and central white matter tracts. Diffusion imaging has already shown evidence of white matter atrophy in the TG of patients with chronic ocular pain.^216^ Functional activation can be studied with event-related functional MRI (fMRI), which uses blood oxygen level–dependent (BOLD) signals as indirect measures of neural activity during stimulus presentation. Recently, event-related fMRI has demonstrated that the trigeminal nociceptive system may also contribute to pain and photophobia in patients with a corneal abrasion^163^^,^^217^ and patients with chronic ocular pain.^163^^,^^217^^,^^218^ Resting-state fMRI assesses functional connectivity of brain networks independent of stimulus events, useful for assessing systemic processes and psychological factors related to pain. Resting-state fMRI has revealed that central brain mechanisms, particularly within the trigeminal system, contribute as much, and sometimes more, to chronic ocular pain symptoms compared with ocular surface pathology.^219^ These measures will help bridge current corneal nerve health assessment with patient symptoms and treatment outcomes.

The integration of peripheral and central imaging approaches offers powerful insights into ocular pain mechanisms. IVCM provides peripheral biomarkers of the peripheral nervous system, diffusion MRI analyzes microstructural integrity across the trigeminal pathway, and fMRI links patient-reported symptoms with brain activity patterns. Parallel studies can be performed in mice with IVCM or multi-photon imaging with endpoint brain activity markers or similar in vivo brain imaging methods, enhancing the opportunity for discovery and cross-species validation. Future studies harmonizing IVCM metrics, characterizing diffusion MRI and fMRI findings, and correlating both approaches are necessary to better characterize patients, guide targeted treatment, and monitor recovery.

Improved integration of preclinical and clinical imaging approaches will enhance mechanistic understanding. Novel imaging techniques combined with genetic tools will enable longitudinal studies of disease progression and cellular mechanisms of corneal regeneration in real time with the means to monitor real-time activity^220^^–^^223^ over large fields of view.^224^^–^^226^ With new optogenetic tools,^225^ cell type–specific and temporally precise perturbation of specific corneal nerves in both homeostatic and disease models can be correlated to their roles in lacrimation, blink reflexes, epithelial homeostasis, and pain perception. These developing modalities will bring tighter connections between animal studies and clinical practice, with the combination of functional imaging and antibody staining for specific nerve receptors potentially enabling nerve fiber typing based on morphology alone to inform clinical IVCM and OCT observations.

## Conclusions: A Roadmap for Advancing Understanding of Corneal Neurobiology

**Figure 3.**
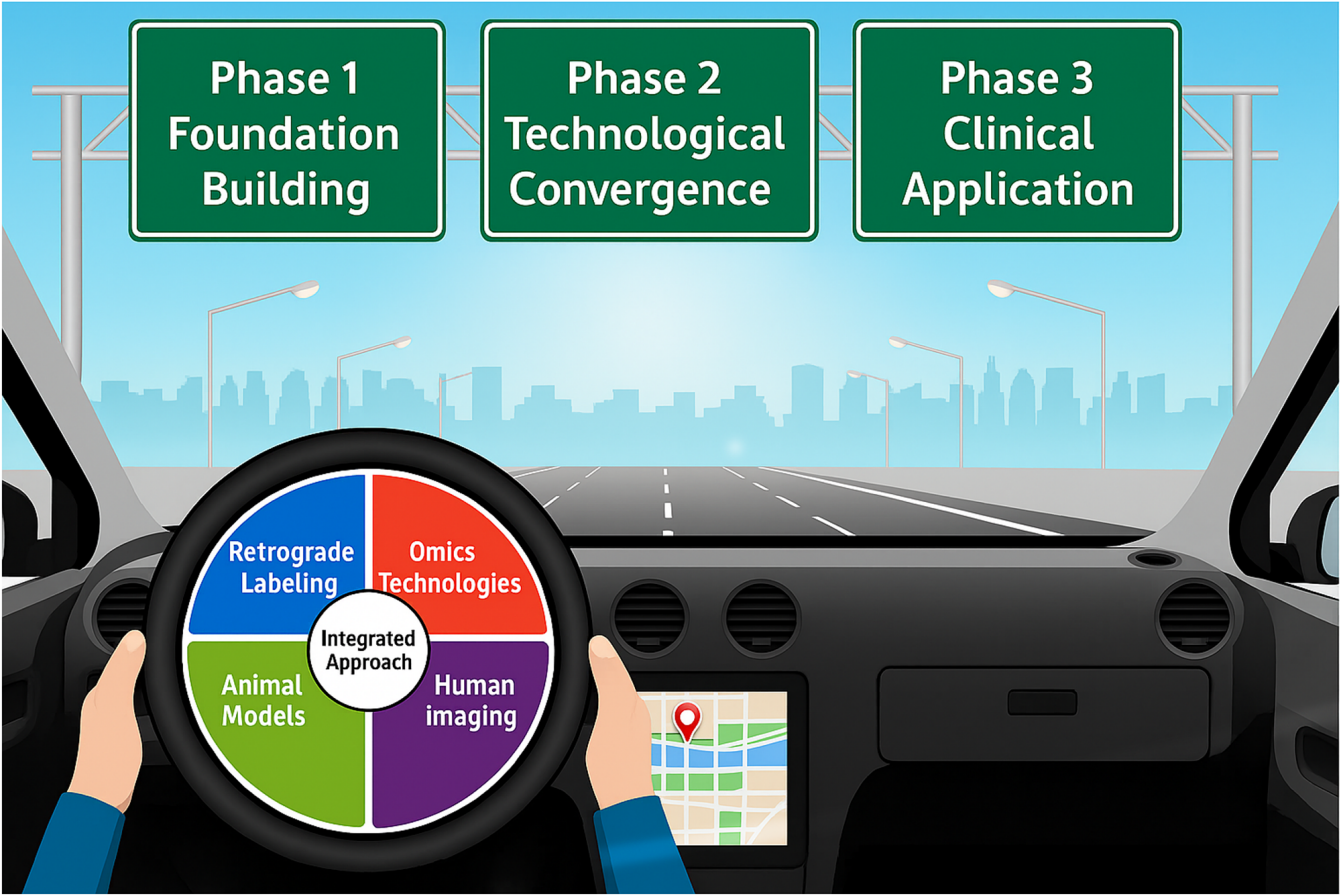
**Three-phase roadmap for corneal innervation research.** The consortium roadmap integrates four technological pillars—retrograde labeling (*blue*), omics (*red*), animal models (*green*), and clinical imaging (*purple*)—through three phases: Phase 1 establishes standardized protocols and quality metrics, phase 2 achieves technological convergence through multimodal integration, and phase 3 advances clinical application via therapeutic target identification and precision medicine implementation. This systematic integration demonstrates how coordinated multi-institutional collaboration accelerates discovery and clinical translation.

Since the 2010 NEI workshop identified critical gaps in corneal pain and sensation research, technological breakthroughs have finally positioned the field to address longstanding challenges. The National Eye Institute's U01-funded consortium on ocular surface innervation harnesses these advances through systematic integration of four complementary pillars: retrograde labeling, omics technologies, animal models, and clinical imaging. This roadmap demonstrates how coordinated multi-institutional collaboration synthesizes diverse approaches into mechanistic frameworks guiding therapeutic development across three phases: foundation building, technological convergence, and clinical translation (Fig. 3).

### Near-Term Priorities: Foundation Building

Immediate priorities establish reproducible methodological foundations across all four approaches. Retrograde labeling will undergo optimization of protocols to review tracer type and standardization of delivery methods enabling cross-laboratory comparisons of quality control metrics. This standardization will enable the current development of harmonized atlases to incorporate retrograde tracing with single-cell transcriptomics and create the first tissue-specific neuronal reference standards. Separately, application of expanded behavioral quantification in animal models will be harmonized, with efforts to incorporate and expand AI-assisted analyses to standardize pain assessment across sites and reduce investigator bias. Finally, clinical imaging is likewise undergoing protocol harmonization for image acquisition and automated analysis pipelines to address reproducibility challenges that have limited clinical adoption. These standardization efforts across the four pillars will help define best practices that accelerate progress across the field by enabling meaningful cross-laboratory and cross-species comparisons.

### Emerging Capabilities: Technological Convergence

The intermediate phase emphasizes technological convergence that transcends individual limitations by integrating unique strengths through complementary approaches. Clinical imaging will combine peripheral IVCM with central fMRI and diffusion MRI to map pain pathways from cornea to brain. Going one step further, such human MRI mapping can potentially be correlated with activity-dependent labeling systems in animal models that map neural circuits responding to corneal injury, helping bridge animal models and clinical populations. With respect to animal models, advanced methodologies such as optogenetic and chemogenetic manipulations will enable precise temporal control of corneal afferents paired with real-time behavioral and neurophysiology recordings for longitudinal tracking of corneal innervation dynamics. Meanwhile, spatial transcriptomic technologies will reveal how neuronal subtypes organize within TG and interact with immune populations, providing unprecedented resolution of neuroimmune networks. When partnered with single-cell (or nucleus) RNA-seq and retrograde labeling, such technologies will generate high-dimensional taxonomies of corneal afferent subtypes, revealing regulatory networks controlling tissue-specific innervation patterns. Animal models will further incorporate comprehensive epithelial and immune cell omic profiling alongside afferent analysis, identifying ligand–receptor pairs mediating neuro-surface crosstalk in health and disease. Furthermore, cross-species transcriptomic profiling will establish “corneal signatures” enabling inference of human neuronal identity from precise animal studies. The convergence of these four pillars is designed to answer questions that none can resolve alone: identifying which neuronal populations are affected in disease, defining their molecular signatures, establishing causal mechanisms, and validating those mechanisms in clinical populations.

### Advancing Toward Clinical Translation

Translating mechanistic insights from this integrated approach toward clinical applications will require harmonized cross-species datasets to identify conserved therapeutic targets while accounting for species-specific molecular differences. Understanding corneal innervation requires examining how sensory neurons maintain ocular surface homeostasis while processing environmental signals. As demonstrated throughout this roadmap, corneal nerves regulate epithelial cell division and migration, coordinate neuroimmune interactions, control protective reflexes including blinking and tearing, and mediate sensory experiences. Disruptions of these functions manifest across a spectrum of clinical conditions: Patients with neurotrophic keratitis face vision-threatening epithelial breakdown from loss of nerve trophic support; DED involves both homeostatic disruption and altered corneal sensation; viral infections such as HSV-1 cause nerve damage with the potential of regeneration; and neuropathic ocular pain represents dysfunction in sensory processing pathways. Clinical correlation studies linking IVCM metrics with diffusion MRI and fMRI findings reveal how dysfunction at any level, from neuroimmune–epithelial interactions to central processing, contributes to disease pathology, enabling patient characterization, targeted treatment selection, and recovery monitoring.

### Consortium Paradigm

This consortium roadmap demonstrates how coordinated, multi-institutional research accelerates discovery through systematic technology integration rather than isolated advancement. The consortium approach establishes data-sharing platforms, standardized protocols, and cross-site validation studies that maximize the potential of each technology while minimizing individual limitations, providing a template for addressing complex biological systems requiring integration across molecular, cellular, circuit, and clinical levels. Notably, many investigations employ parallel questions, assays, and analyses between animal models and human clinical presentation, reflecting the historic and current concerted effort to understand both the biology of corneal innervation and human disease. By establishing corneal innervation as a model system for understanding peripheral neural control in health and disease, this roadmap positions the field to unlock therapeutic potential not only for ocular surface disorders but also for broader applications in pain medicine, regenerative neuroscience, and precision healthcare.

The clinical urgency is clear. Patients experiencing persistent postoperative pain after LASIK or cataract surgery or post-herpetic neuralgia face lasting and debilitating symptoms.^227^ Those with chronic DED, NCP, and neurotrophic keratitis experience substantial quality of life impairment, with symptoms interfering with driving, reading, social engagement, and work productivity. Standardized behavioral assessments integrated with molecular circuit definitions will accelerate development of precision analgesics and regenerative therapies that selectively restore homeostatic function, promote nerve regeneration, or modulate sensory processing while preserving essential corneal functions. Crucially, this vision is only achievable through the efforts of a sustained, cross-disciplinary team of ophthalmologists, neuroscientists, pain researchers, immunologists, imaging experts, engineers, and computational biologists working together with shared protocols, data standards, and validation pipelines. The systematic integration of complementary technologies described here demonstrates how coordinated multi-institutional expertise can address longstanding clinical challenges while establishing new paradigms for treating diseases of the nervous system.
